# Wider means worsen? Influence of QRS duration of left bundle branch block on prognosis of patients after transcatheter aortic valve replacement

**DOI:** 10.1097/MD.0000000000041940

**Published:** 2025-03-28

**Authors:** Jiajun Pan, Zhimin Zhao, Bailing Li, Hao Zhang, Chengliang Cai, Yun Tao, Fan Qiao, Fanglin Lu, Lin Han, Zhiyun Xu

**Affiliations:** a Department of Cardiovascular Surgery, The First Affiliated Hospital of Naval Medical University, Shanghai, People’s Republic of China.

**Keywords:** left bundle branch block, left ventricular ejection fraction, transcatheter aortic valve replacement

## Abstract

The impact of QRS duration on postoperative LBBB and its implications for the prognosis of patients undergoing transcatheter aortic valve replacement (TAVR) remained uncertain. This study enrolled consecutive patients who underwent TAVR with self-expanding prostheses in our department from September 2017 to January 2021. Based on the pro-discharge electrocardiogram, patients were categorized into 3 groups: Group-NCD (no conduction disorder), Group-sLBBB (LBBB, QRS ≥ 150 ms), and Group-mLBBB (LBBB, QRS < 150 ms). Basic characteristics were compared among these groups. Furthermore, differences in left ventricular ejection fraction (LVEF), survival rates, and clinical events were assessed at baseline, discharge, and during a one-year follow-up period. A total of 56 patients were included in the study. With 17 (30.36%) experiencing new-onset LBBB, of which eleven had a QRS duration ≥ 150 ms. Group-sLBBB exhibited a longer left ventricular end-diastolic diameter at baseline. At a one-year follow-up, the LVEF improved in Group-NCD, but not in the LBBB groups. At discharge, the LVEF of Group-sLBBB was lower than that of Group-NCD (52.82 ± 11.48 vs 61.48 ± 10.10, *P* = .036) and remained lower at follow-up (57.10 ± 9.49 vs 65.85 ± 7.58, *P* = .011). Additionally, the LVEF of Group-sLBBB was lower than that of Group-mLBBB at discharge (52.82 ± 11.48 vs 63.17 ± 4.31, *P* = .018). However, there were no significant differences in survival and event-free survival among the groups. The study revealed a notable occurrence of new-onset LBBB following TAVR, with a majority of cases exhibiting a significantly prolonged QRS duration (≥150 ms). While the presence of LBBB did not impact one-year survival or clinical events, it did exert adverse effects on LVEF. Notably, when QRS duration was markedly prolonged, these adverse effects manifested earlier and were more pronounced.

**Key points**

- The majority of new-onset left bundle branch block (LBBB) following transcatheter aortic valve replacement (TAVR) is characterized by a significantly prolonged QRS duration (QRS ≥ 150 ms).
- LBBB, in general, does not exert an impact on 1-year survival or clinical events.
- LBBB with seriously prolonged QRS has early and obviously adverse effects on LVEF.
- LBBB is a frequent complication of TAVR. The impact of LBBB on mortality post-TAVR remains a subject of controversy, although the consensus from most studies indicates that LBBB tends to influence left ventricular function.
- The majority of cases involving new-onset LBBB after TAVR are characterized by a significantly prolonged QRS duration (QRS ≥ 150 ms).
- Notably, when LBBB is accompanied by a seriously prolonged QRS duration, it exhibits early and evident adverse effects on LVEF.
- Pay more attention to heart function of the patient with new-onset LBBB with seriously prolonged QRS duration after TAVR.
- To the patient with seriously prolonged QRS duration after TAVR and reduced LVEF, CRT should be considered.

## 1. Introduction

Since the inception of transcatheter aortic valve replacement (TAVR) in 2002,^[1]^ the indications for TAVR have undergone continuous expansion. Despite advancements in prosthetic technology and the increasing expertise of operators, the incidence of complications, such as moderate to severe paravalvular leakage, has progressively diminished. However, arrhythmia, particularly left bundle branch block (LBBB) or atrioventricular block (AVB), remain the most prevalent complications of TAVR. Notably, the frequency of these arrhythmias has not significantly decreased even with the ongoing updates to TAVR devices.^[2,3]^ The impact of LBBB on the prognosis of patients remains a subject of debate. The duration of the QRS complex on the electrocardiogram (ECGs) serves as an indicator of ventricles ventricular synchrony. Uncertainty persists regarding whether the QRS duration of postoperative LBBB has any consequential effect on the cardiac function and prognosis of patients undergoing TAVR. The primary objective of this study was to assess the occurrence of new-onset LBBB following TAVR with self-expanding prostheses and to investigate the influence of LBBB with varying degrees of QRS duration on the prognosis of these patients.

## 2. Methods

### 2.1. Study population

The retrospective study consecutively enrolled 81 patients admitted between September 1, 2017, to January 31, 2021, presenting with severe aortic stenosis or regurgitation. These patients met the indications for TAVR, which included either surgical contraindications or a high-risk surgery status determined by the Society of Thoracic Surgeon score. The selected individuals underwent transfemoral artery TAVR using self-expanding prostheses in our department. This study was reviewed and approved by the First Affiliated Hospital of Naval Medical University ethical review board.

Exclusion criteria comprised a history of permanent cardiac pacemaker implantation before surgery, preoperative LBBB, transapical TAVR, intraoperative conversion to cardiopulmonary bypass thoracotomy, or mortality during hospitalization. The prostheses utilized in the TAVR procedures included Venus-A prostheses (manufactured by Hangzhou Qiming Medical Instrument Co., Ltd., Hangzhou, China) and VitaFlow prostheses (manufactured by Shanghai MicroPort Medical Technology Co., Ltd., Shanghai, China).

### 2.2. Data collection

General information, medical history, and preoperative laboratory tests, including blood routine, hepatic and renal function, fasting blood glucose, and brain natriuretic peptide, were meticulously recorded for all patients at baseline. Additionally, 12-lead ECGs and echocardiographies were performed at baseline and predischarge.

**Figure 1.**
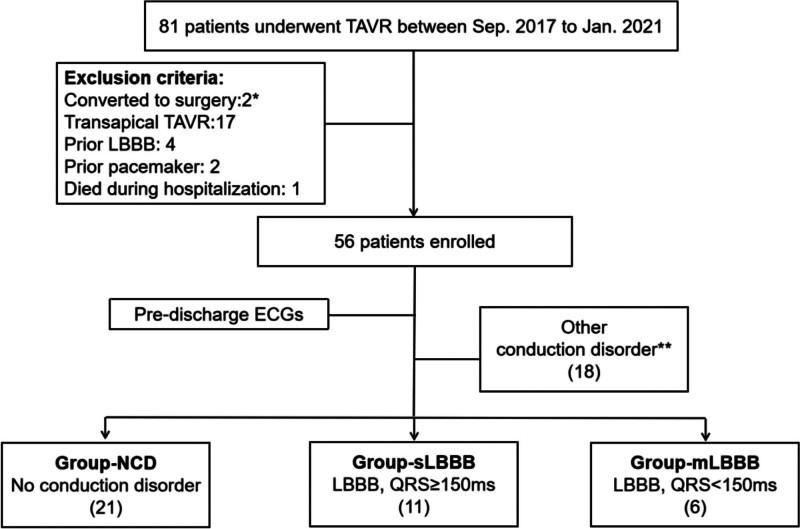
Flow chart of patient enrollment. ECG: Electrocardiogram; mLBBB: mildly prolonged left bundle branch block with QRS duration < 150 ms; NCD: no conduction disorder; LBBB: Left bundle branch block; sLBBB: seriously prolonged left bundle branch block with QRS duration ≥ 150ms; TAVR: Transcatheter aortic valve replacement. * include 1 transapical TAVR; ** Other conduction disorder included conduction disorders such as AVB, right bundle branch block (RBBB), intraventricular block and fascicular block. AVB = atrioventricular block.

Based on the predischarge ECGs, patients were categorized into 3 groups: Group-NCD (no conduction disorder), Group-sLBBB (QRS duration was seriously prolonged, with QRS ≥ 150 ms), and Group-mLBBB (QRS duration was mildly prolonged, with QRS < 150 ms) as illustrated in Fig. 1. Patients exhibiting other conduction disorders such as AVB, right bundle branch block (RBBB), intraventricular block, and fascicular block, which could potentially interfere with the results, were excluded from statistical analysis.

Baseline characteristics, preoperative laboratory tests, and echocardiography were compared among Group-NCD, Group-sLBBB, and Group-mLBBB.

Patients underwent a follow-up period averaging 1 year, during which adverse events including death, pacemaker (PPM) implantation, and readmission were meticulously recorded. Echocardiography data were also reviewed during this follow-up period.

Image acquisition using two-dimensional echocardiography (2D Echo) ensures that cross-sectional images of the left ventricle are obtained in a multi-plane (usually four-chamber and long-axis) position. Simpson biplane method was used to calculate the volume of the left ventricle by drawing the intima contour of the left ventricle during systolic and diastolic periods, thus obtaining left ventricular ejection fraction (LVEF). The formula is calculated as follows:


$$\begin{array}{l} LVEF=\left(EDV-ESV\right)/EDV\times 100\% \end{array}$$

LVEF at baseline, discharge, and follow-up were compared within each group and acorss the 3 groups. Furthermore, one-year survival and event-free survival were assessment and compared among the 3 groups.

### 2.3. Statistical methods

Statistical analysis was conducted utilizing SPSS 25.0 software. Normally distributed measurement data were presented as mean ± standard deviation, while non-normally distributed measurement data were expressed as the median [interquartile range]. Enumeration data were presented as cases and percentages.

For comparisons between groups, the independent samples t-test or rank sum test was employed for measurement data. The paired t-test and related samples rank sum test were utilized to compare LVEF at baseline, discharge, and one-year follow-up within each group. The Fisher exact test was applied for the comparison of enumeration data.

Survival and event-free survival were calculated and presented using Kaplan–Meier curves. A significance level of *P* < .05 was considered indicative of statistical significance.

## 3. Results

### 3.1. Incidence and baseline parameters of new-onset LBBB

A total of 56 patients were included in the study. Analysis of baseline and predischarge ECGs revealed that seventeen patients (30.36%) developed new-onset LBBB postoperatively, with eleven cases (64.71%) exhibiting a QRS duration ≥ 150 ms.

At baseline, the left ventricular end-diastolic diameter was observed to be longer in Group-sLBBB compared to Group-NCD (5.53 ± 0.93 vs 4.75 ± 0.99 cm, *P* = .038). However, no significant differences were noted in basic characteristics, preoperative laboratory tests results, and other echocardiographic parameters as detailed in Table 1.

**Table 1:** Baseline characteristics.

	Group-NCD (n = 21)	Group-sLBBB (n = 11)	Group-mLBBB (n = 6)	*P* value*	*P* value**	*P* value***
Age (yrs)	75.33 ± 6.21	77.45 ± 5.11	74.67 ± 5.24	.339	.813	.303
Age[median (25%-75%)]	74.53 (69.09–78.88)	73.34 (72.47–77.12)	76.76 (74.32–79.81)			
Female (%)	12 (57.14)	6 (54.55)	3 (50.00)	1.000	1.000	1.000
CHD (%)	5 (23.81)	5 (45.45)	1 (16.67)	.252	1.000	.333
PCI (%)	2 (9.52)	2 (18.18)	0 (0.00)	.593	1.000	.515
Diabetes mellitus (%)	4 (19.05)	1 (9.09)	0 (0.00)	.637	.545	1.000
Hypertension (%)	13 (61.90)	10 (90.91)	3 (50.00)	.115	.662	.099
Stroke (%)	1 (4.76)	1 (9.09)	0 (0.00)	1.000	1.000	1.000
Syncope (%)	4 (19.05)	1 (9.09)	1 (16.67)	.637	1.000	1.000
TBil (umol/L)	11.49 ± 4.17	13.56 ± 6.36	13.72 ± 6.46	.274	.317	.963
ALT (U/L)	13.00 (10.00,23.50)	12.00 (9.00,21.00)	15.50 (11.75,38.25)	.431	.720	.460
AST (U/L)	20.00 (15.50,23.50)	18.00 (13.00,22.00)	19.50 (16.00,33.25)	.563	.988	.504
Albumin (g/L)	38.67 ± 3.34	39.09 ± 2.77	37.33 ± 3.14	.721	.391	.251
FBG (mmol/L)	5.60 ± 1.28	5.66 ± 0.98	6.58 ± 3.69	.894	.545	.569
SCr (umol/L)	80.00 (71.00,100.50)	75.00 (66.00,98.00)	86.00 (76.75,141.50)	.618	.467	.575
BUN (mmol/L)	8.42 ± 4.36	7.47 ± 2.96	8.92 ± 6.01	.524	.822	.599
Hemoglobin (g/L)	115.19 ± 25.36	121.45 ± 12.75	116.17 ± 17.87	.360	.931	.488
Platelet (×10^9^/L)	185.29 ± 99.35	184.91 ± 55.96	163.00 ± 39.64	.991	.600	.412
BNP (pg/ml)	813.22 ± 1240.14	796.75 ± 821.94	861.72 ± 1649.93	.969	.939	.914
NYHA (%)						
Class II	3 (14.29)	1 (9.09)	2 (33.33)	1.000	.555	.515
Class III	15 (71.42)	6 (54.55)	2 (33.33)	.442	.153	.620
Class IV	3 (14.29)	4 (36.36)	2 (33.33)	.197	.555	1.000
Length of stay in hospital (d)	20.71 ± 12.37	25.45 ± 12.49	27.17 ± 31.42	.313	.642	.873
LVEDD (cm)	4.75 ± 0.99	5.53 ± 0.93	5.08 ± 0.26	.038	.468	.316
LVEF (%)	59.05 ± 13.76	56.36 ± 10.23	59.67 ± 15.69	.574	.926	.605
Bicuspid aortic valve(%)	11 (52.38)	4 (36.36)	2 (33.33)	.472	.648	.620
Mitral regurgitation (ml)	2.80 ± 4.39	6.24 ± 5.48	8.10 ± 10.19	.063	.314	.636
Ascending aorta diameter (cm)	3.70 ± 0.47	3.92 ± 0.78	3.72 ± 0.51	.341	.934	.615
AV annulus diameter (cm)	2.14 ± 0.25	2.10 ± 0.32	2.13 ± 0.10	.687	.894	.881
Aortic root diameter (cm)	2.13 ± 0.24	2.10 ± 0.32	2.14 ± 0.09	.740	.952	.789

ALT = alanine aminotransferase; AST = aspartate aminotransferase; BNP = brain natriuretic peptide; BUN = blood urea nitrogen; CHD = coronary heart disease; FBG = fasting blood glucose; LVEDD = left ventricular end-diastolic diameter; LVEF = left ventricular ejection fraction; mLBBB = mildly prolonged left bundle branch block with QRS duration < 150 ms; NCD = no conduction disorder; PCI = percutaneous coronary intervention; SCr = serum creatine; sLBBB = seriously prolonged left bundle branch block with QRS duration ≥ 150 ms; TBil = total bilirubin.

* *P* Group-NCD vs. Group-sLBBB.

** *P* Group-NCD vs. Group-mLBBB.

*** *P* Group-sLBBB vs. Group-mLBBB.

### 3.2. Impact of new-onset LBBB on LVEF

**Figure 2.**
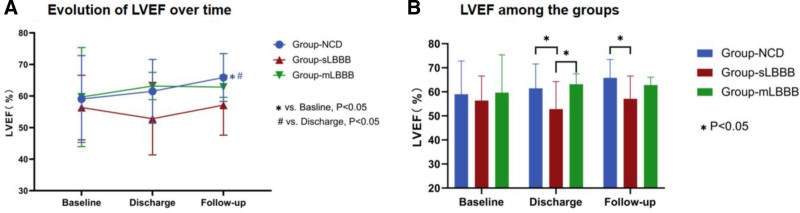
(A) Evolution of LVEF over time. (B) LVEF at baseline, discharge, 1-year follow-up among the 4 groups. mLBBB: mildly prolonged left bundle branch block with QRS duration < 150 ms; NCD = no conduction disorder; LVEF = left ventricular ejection fraction; sLBBB = seriously prolonged left bundle branch block with QRS duration ≥ 150 ms.

At one-year follow-up, LVEF in Group-NCD demonstrated an increase compared to baseline and discharge (follow-up vs baseline: 65.85 ± 7.58% vs 59.05 ± 13.76%, *P* = .013, follow-up vs discharge: 65.85 ± 7.58% vs 61.48 ± 10.10%, *P* = .023) (Fig. 2A). Conversely, there were no significant changes in LVEF for Group-sLBBB (follow-up vs baseline: 57.10 ± 9.49% vs 56.36 ± 10.23%, *P* = .769, follow-up vs discharge: 57.10 ± 9.49% vs 52.82 ± 11.48%, *P* = .256) and Group-mLBBB (follow-up vs baseline: 62.83 ± 3.25% vs 59.67 ± 15.69%, *P* = .655, follow-up vs discharge: 62.83 ± 3.25% vs 63.17 ± 4.31%, *P* = .849).

While there was no significant differences in baseline LVEF among Group-NCD, Group-sLBBB, and Group-mLBBB, at discharge, LVEF in Group-sLBBB was lower compared to Group-NCD and Group-mLBBB (Group-sLBBB vs Group-NCD: 52.82 ± 11.48% vs 61.48 ± 10.10%, *P* = .036, Group-sLBBB vs Group-mLBBB: 52.82 ± 11.48% vs 63.17 ± 4.31%, *P* = .018). At the one-year follow-up, LVEF in Group-sLBBB remained lower than Group-NCD (Group-sLBBB vs Group-NCD: 57.10 ± 9.49% vs 65.85 ± 7.58%, *P* = .011) (Fig. 2B).

### 3.3. Impact on survival and clinical events

**Figure 3.**
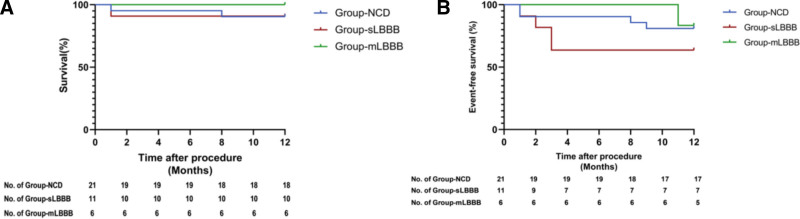
(A) Kaplan–Meier curves are displayed for 1-year survival. (B) Kaplan–Meier curves are displayed for 1-year event-free survival. mLBBB = mildly prolonged left bundle branch block with QRS duration < 150 ms; NCD = no conduction disorder; sLBBB = seriously prolonged left bundle branch block with QRS duration ≥ 150 ms.

During the one-year follow-up period, within Group-NCD, 2 patients experienced mortality (1 due to malignant tumor, 1 due to pulmonary infection), and 2 patients were readmitted to the hospital (1 for cerebral infarction, the other for pulmonary infection). In Group-sLBBB, 1 patient succumbed to myocardial infarction and malignant arrhythmia, and 3 patients were readmitted to the hospital (2 for heart failure and 1 for syncope). Notably, 1 patient in Group-mLBBB was readmitted for PPM implantation due to bradycardia. There were no significant differences in survival (Fig. 3A) and event-free survival (Fig. 3B) among the 3 groups (*P* > .05).

## 4. Discussion

### 4.1. Incidence of new-onset LBBB after TAVR

New-onset LBBB is the most common conduction abnormality after TAVR,^[2,4,5]^ with an incidence ranging from 4% to 65% among patients.^[2]^ This wide variability suggests that various factors may influence the occurrence of LBBB in different individuals, such as the implantation of a Medtronic CoreValve (vs Edwards SAPIEN valves), depth of implantation, overexpansion of the native aortic annulus, and larger valve size. It is noteworthy that, despite the lack of a significant reduction in incidence with the use of new-generation valves,^[2,3]^ we have observed the persistent presence of this phenomenon. Consistent with findings in other studies, the occurrence of postoperative new-onset LBBB in this study was 30.36%. Notably, the majority of these cases (64.71%) exhibited LBBB with a QRS complex duration ≥ 150 ms.

### 4.2. Causes and risk factors of new-onset LBBB after TAVR

The close anatomical proximity^[6]^ during TAVR, particularly in the processes of wire insertion, valve implantation, and balloon dilating, can potentially impact the left bundle branch block. Mechanical damage to the conduction system may occur directly from the expanded prosthesis, resulting in edema, hematoma, and ischemia to varying degrees,^[7]^ ultimately leading to the development of LBBB. Furthermore, the adjacent of the aortic valve may contribute to calcium deposits in the conduction bundle. These calcium deposits, coupled with the deterioration of left ventricular function, may be associated with the onset or exacerbation of LBBB in patients with aortic stenosis/regurgitation.^[3]^

The depth of prosthesis implantation in the left ventricular outflow tract is widely acknowledged as a significant risk factor for the emergence of new-onset LBBB following TAVR.^[3,8,9]^ Additionally, studies have reported that, in comparison to the Sapien prosthesis, the Corevalve prosthesis has a higher incidence of LBBB.^[10–12]^ However, there is ongoing controversy surrounding other contributing factors.

Some studies have suggested associations with prior first-degree AVB and the duration of the baseline QRS complex,^[8,13]^ prior coronary artery bypass grafting,^[14]^ female gender,^[12]^ degree of calcification,^[13]^ prosthesis size,^[10]^ annulus overexpansion,^[9,14]^ repositioning, late radial expansion,^[15]^ and various other factors. In the current study, we observed a longer left ventricular end-diastolic diameter in Group-sLBBB, suggesting a potential association between left ventricular enlargement and the development of new-onset LBBB with a significantly widened QRS duration after TAVR in patients with aortic stenosis or regurgitation.

### 4.3. Effects of LBBB on the prognosis of patients after TAVR

#### 4.3.1. Mortality

The impact of LBBB on mortality following TAVR remains a subject of controversy, as observed in various studies.^[11,12,14,16–21]^ Some investigations have reported an association between new-onset LBBB after TAVR and increased mortality.^[11,12,20,21]^ Furthermore, certain studies have proposed that the duration of the QRS complex is an independent predictor of all-cause mortality.^[22]^ On the contrary, several studies have found no significant increase in mortality associated with LBBB after TAVR.^[14,16–19]^ A meta-analysis incorporating 8 studies with 4756 patients concluded LBBB after TAVR increased the risk of cardiac death (RR 1.39; 95% CI 1.04–1.86) but did not significantly impact all-cause mortality (RR 1.21; 95% CI 0.98–1.50).^[4]^

The factors contributing to an increased risk of death^[4,23]^ are primarily attributed t: (1) patients with new-onset LBBB after TAVR being susceptible to late complete AVB, which carries the potential for sudden cardiac death; (2) the cardiac asynchronous induced by LBBB may progressively exacerbate heart failure, consequently raising cardiovascular mortality. However, it is essential to acknowledge that the older age of the included population, coupled with preoperative comorbidities, high surgical risk, and elevated noncardiac mortality, might have obscured the true impact of LBBB.

In this study, 3 patients died in follow-up period (one for malignant tumor, 1 for pulmonary infection and 1 for myocardial infarction and malignant arrhythmia). While we observed that the persistent presence of LBBB after TAVR did not have a statistically significant impact on one-year survival rates or event-free survival rates, it is noteworthy that the overall mortality rate approached 5%, indicating a relatively elevated level in this patient cohort. It is essential to emphasize that this higher mortality rate may be influenced by various factors, including preexisting comorbidities in patients before surgery, postoperative complications, and challenges during the postoperative recovery process. We conducted a thorough analysis of the causes of these mortality events and confirmed that these events were not attributable to TAVR technology itself but could be the result of a comprehensive impact of the patients’ overall health condition and other factors. This includes, but is not limited to, preexisting health issues and postoperative recovery progress. Therefore, TAVR technology itself is considered qualified.

#### 4.3.2. Heart failure

Currently, the prevailing consensus from various studies suggests that LBBB exerts an impact on the left ventricular function of patients post TAVR.^[14,16,18,20,24,25]^ This study aligns with these findings, where the long-term follow-up, spanning from 6 months to 3 years, consistently reveals lower LVEF in LBBB patients compared to their non-LBBB counterparts. Baseline LVEF did not exhibit differences among the groups in this study. However, at the one-year follow-up, patients without conduction disorder (Group-NCD) demonstrated an improvement in LVEF, contrasting with patients with LBBB who did not show significant improvement. Particularly noteworthy were observations in LBBB patients in Group-sLBBB, revealing more pronounced and earlier influence on LVEF. Their LVEF was significantly lower than that of Group-NCD patients at discharge and follow-up, and also lower than that of Group-mLBBB patients at discharge. Interestingly, Group-mLBBB exhibited no significantly difference in LVEF compared to Group-NCD at discharge or one-year follow-up. These findings could explained by the abnormal electrical activity associated with LBBB leading to mechanical asynchrony within the left and right ventricles, as well as within the left ventricle itself.^[26]^ This process contributes to increased ventricular end-systolic volume, septal hypertrophy, adverse remodeling,^[24]^ abnormal blood perfusion, and impaired systolic and diastolic ventricular function.^[27]^ The severity of asynchrony appears to be more pronounced and impactful on cardiac function when the QRS duration is significantly prolonged (QRS ≥ 150 ms).

#### 4.3.3. Management of new-onset LBBB after TAVR

Prophylactic PPM implantation is not deemed appropriate for patients experiencing new-onset LBBB after TAVR.^[28,29]^ In accordance with the 2021 ESC Guidelines on cardiac pacing and cardiac resynchronization therapy^[26]^ concerning the management of conduction abnormalities post-TAVR, persistent new LBBB with QRS duration > 150 ms or PR interval > 240 ms, with no further prolongation beyond 48 hours post-procedure, is suggested to undergo ambulatory ECG monitoring (Class IIa) or electrophysiological study (Class IIa). If electrophysiological study is contemplated, it should be conducted 3 days after surgery and after conduction abnormalities have stabilized.^[26]^ It is crucial to critically evaluate the necessity of pacing in patients with LBBB after TAVR to avoid unnecessary PPM implantation.^[30]^ Presently, the indications of CRT in patients with LBBB after TAVR should adhere to the guidelines,^[26]^ particularly for those with reduced LVEF. In case where postoperative LBBB worsening to high-degree AVB/complete heart block and necessitating pacemaker implantation, direct CRT implantation may be considered when LVEF is <40%.

Our study revealed that while LBBB did not exert a significant impact on one-year survival or clinical events following TAVR, its effectiveness in improving LVEF at 1 year post-TAVR was not as pronounced as observed in patients without conduction disorder. Particularly noteworthy were the findings that LBBB patients with seriously prolonged QRS duration (QRS ≥ 150 ms) experienced earlier and more pronounced adverse effect on LVEF. Their LVEF at discharge was significantly lower than that of patients without conduction disorder or those with LBBB and mildly prolonged QRS duration. Additionally, their LVEF remained significantly lower than that of patients without conduction disorder at the one-year follow-up.

Therefore, in cases where patients exhibit high-degree AVB or complete heart block due to the worsening of LBBB after TAVR and require PPM implantation with a LVEF of ≥40%, the consideration of His bundle pacing may be appropriate to reduce the QRS duration during pacing. This approach has the potential to enhance ventricular synchronization during pacing, thereby possibly improving both short-term and long-term cardiac function in patients who undergo pacing after TAVR. However, it necessitates further investigation to establish its efficacy conclusively.

In our study, LVEF in patients with Group-sLBBB was approximately 9.66% lower than that of Group-NCD at discharge and remained approximately 8.75% lower at one-year follow-up, both of which exceeded the threshold of clinical significance, suggesting that LBBB, especially severe LBBB, has a significant adverse effect on cardiac function. The changes of LVEF have important reference value for making individualized treatment plan. For example, patients with a significant decline in LVEF may need more aggressive medication, lifestyle interventions, or even consideration of cardiac resynchronization therapy (CRT) to improve heart function. In addition, persistently low LVEF may suggest the need for closer follow-up and monitoring to prevent and promptly manage heart failure-related complications. Although there was some variation (up to 14%) in LVEF measurements between evaluators, our study managed to limit the actual measurement variation to <5% by using dual evaluator independent measurements and introducing a third evaluator review mechanism (ICC = 0.95, Bland-Altman analysis showed the deviation to be within ± 5%). This rigorous measurement ensures the authenticity and reliability of the LVEF differences, further supporting the clinical relevance of our findings. Even after accounting for measurement errors, the LVEF differences between Group-sLBBB and the other groups were significant and clinically significant.

### 4.4. Limitations

This study, being a single-center investigation, is constrained by its relatively small sample size. Additionally, in patients with essentially normal LVEF, the effects of LBBB on clinical outcome might necessitate longer observation periods. Nonetheless, our findings highlight that LBBB patients, particularly those with a QRS duration ≥ 150 ms, exhibit adverse effects on LVEF after TAVR. In this study, the factors predicting the risk of LBBB were primarily derived from patient general information, medical history, preoperative laboratory tests, and echocardiography, with intraoperative factors not being included. The use of different prostheses may present varying risks of LBBB. While efforts were made to mitigate the impact of prostheses by employing self-expanding prostheses through the femoral artery, there may still be subtle differences among prostheses that necessitate further investigation. In practical clinical application, the measurement of LVEF still has some variability.^[31]^ Factors such as the experience level of different evaluators, equipment performance and image quality may affect the accuracy of measurement results, and thus affect the universality of research conclusions.

Future studies should consider multicenter, large sample size designs to improve the external validity and generalization of the results. At the same time, the use of multiple assessment tools (such as 3D echocardiography, CMR) to measure LVEF, and analysis of intra-observer and inter-observer variability, will help further validate the accuracy of LVEF assessment. In addition, extended follow-up and comprehensive documentation of intraoperative details and the effects of different prosthesis types will contribute to a more comprehensive understanding of the impact of LBBB on LVEF and the patient’s long-term prognosis.

## 5. Conclusions

The incidence of new-onset LBBB after TAVR was notably high, predominantly characterized by LBBB with QRS duration ≥ 150ms. Left ventricular enlargement may be associated with the occurrence of postoperative new-onset LBBB. While the presence of LBBB did not significantly impact one-year survival or clinical events following TAVR, it exhibited limited effectiveness in improving LVEF at 1 year compared to cases without conduction disorders. Notably, in instances of severely prolonged QRS duration (≥150 ms), detrimental effects on LVEF became evident earlier and more prominently.

## Author contributions

**Conceptualization:** Jiajun Pan, Zhimin Zhao, Chengliang Cai, Yun Tao, Fan Qiao, Fanglin Lu, Lin Han, Hao Zhang, Zhiyun Xu, Bailing Li.

**Data curation:** Jiajun Pan, Zhimin Zhao, Chengliang Cai, Fan Qiao, Fanglin Lu, Lin Han, Hao Zhang, Zhiyun Xu, Bailing Li.

**Formal analysis:** Fan Qiao, Fanglin Lu, Lin Han, Bailing Li.

**Funding acquisition:** Hao Zhang, Zhiyun Xu.

**Investigation:** Yun Tao, Jiajun Pan, Zhimin Zhao.

**Methodology:** Yun Tao, Jiajun Pan, Hao Zhang, Zhiyun Xu, Bailing Li.

**Writing – original draft:** Jiajun Pan, Zhimin Zhao, Chengliang Cai, Yun Tao, Fan Qiao, Fanglin Lu, Lin Han, Hao Zhang, Zhiyun Xu, Bailing Li.

**Writing – review & editing:** Jiajun Pan, Zhimin Zhao, Chengliang Cai, Fan Qiao, Hao Zhang, Zhiyun Xu, Bailing Li.

## References

[1] Cribier A Eltchaninoff H Bash A. Percutaneous transcatheter implantation of an aortic valve prosthesis for calcific aortic stenosis: first human case description. Circulation. 2002;106:3006–8. 12473543 10.1161/01.cir.0000047200.36165.b8

[2] Auffret V Puri R Urena M. Conduction disturbances after transcatheter aortic valve replacement: current status and future perspectives. Circulation. 2017;136:1049–69. 28893961 10.1161/CIRCULATIONAHA.117.028352

[3] Muntané-Carol G Guimaraes L Ferreira-Neto AN. How does new-onset left bundle branch block affect the outcomes of transcatheter aortic valve repair? Expert Rev Med Devices. 2019;16:589–602. 31172837 10.1080/17434440.2019.1624161

[4] Regueiro A Abdul-Jawad Altisent O Del Trigo M. Impact of new-onset left bundle branch block and periprocedural permanent pacemaker implantation on clinical outcomes in patients undergoing transcatheter aortic valve replacement: a systematic review and meta-analysis. Circ Cardiovasc Interv. 2016;9:e003635. 27169577 10.1161/CIRCINTERVENTIONS.115.003635

[5] Rodés-Cabau J Urena M Nombela-Franco L. Arrhythmic burden as determined by ambulatory continuous cardiac monitoring in patients with new-onset persistent left bundle branch block following transcatheter aortic valve replacement: the MARE study. JACC Cardiovasc Interv. 2018;11:1495–505. 30031719 10.1016/j.jcin.2018.04.016

[6] Kawashima T Sato F. Visualizing anatomical evidences on atrioventricular conduction system for TAVI. Int J Cardiol. 2014;174:1–6. 24750717 10.1016/j.ijcard.2014.04.003

[7] Moreno R Dobarro D López de Sá E. Cause of complete atrioventricular block after percutaneous aortic valve implantation: insights from a necropsy study. Circulation. 2009;120:e29–30. 19652115 10.1161/CIRCULATIONAHA.109.849281

[8] Urena M Mok M Serra V. Predictive factors and long-term clinical consequences of persistent left bundle branch block following transcatheter aortic valve implantation with a balloon-expandable valve. J Am Coll Cardiol. 2012;60:1743–52. 23040577 10.1016/j.jacc.2012.07.035

[9] Katsanos S van Rosendael P Kamperidis V. Insights into new-onset rhythm conduction disorders detected by multi-detector row computed tomography after transcatheter aortic valve implantation. Am J Cardiol. 2014;114:1556–61. 25245414 10.1016/j.amjcard.2014.08.020

[10] Bernardi FL Ribeiro HB Carvalho LA. Direct transcatheter heart valve implantation versus implantation with balloon predilatation: insights from the Brazilian transcatheter aortic valve replacement registry. Circ Cardiovasc Interv. 2016;9:e003605. 27496637 10.1161/CIRCINTERVENTIONS.116.003605

[11] Houthuizen P Van Garsse LA Poels TT. Left bundle-branch block induced by transcatheter aortic valve implantation increases risk of death. Circulation. 2012;126:720–8. 22791865 10.1161/CIRCULATIONAHA.112.101055

[12] Schymik G Tzamalis P Bramlage P. Clinical impact of a new left bundle branch block following TAVI implantation: 1-year results of the TAVIK cohort. Clin Res Cardiol. 2015;104:351–62. 25388650 10.1007/s00392-014-0791-2

[13] Hein-Rothweiler R Jochheim D Rizas K. Aortic annulus to left coronary distance as a predictor for persistent left bundle branch block after TAVI. Catheter Cardiovasc Interv. 2017;89:E162–8. 27038099 10.1002/ccd.26503

[14] Nazif TM Williams MR Hahn RT. Clinical implications of new-onset left bundle branch block after transcatheter aortic valve replacement: analysis of the PARTNER experience. Eur Heart J. 2014;35:1599–607. 24179072 10.1093/eurheartj/eht376

[15] Webb JG Sathananthan J. Left bundle branch block and new permanent pacemaker implantation after transcatheter aortic valve replacement: are they benign? JACC Cardiovasc Interv. 2018;11:311–3. 29413245 10.1016/j.jcin.2017.11.021

[16] Urena M Webb JG Cheema A. Impact of new-onset persistent left bundle branch block on late clinical outcomes in patients undergoing transcatheter aortic valve implantation with a balloon-expandable valve. JACC Cardiovasc Interv. 2014;7:128–36. 24440024 10.1016/j.jcin.2013.08.015

[17] Testa L Latib A De Marco F. Clinical impact of persistent left bundle-branch block after transcatheter aortic valve implantation with CoreValve Revalving System. Circulation. 2013;127:1300–7. 23443735 10.1161/CIRCULATIONAHA.112.001099

[18] Chamandi C Barbanti M Munoz-Garcia A. Long-Term outcomes in patients with new-onset persistent left bundle branch block following TAVR. JACC Cardiovasc Interv. 2019;12:1175–84. 31129090 10.1016/j.jcin.2019.03.025

[19] Keßler M Gonska B Seeger J Rottbauer W Wöhrle J. Long-term clinical outcome of persistent left bundle branch block after transfemoral aortic valve implantation. Catheter Cardiovasc Interv. 2019;93:538–44. 30298700 10.1002/ccd.27850

[20] Nazif TM Chen S George I. New-onset left bundle branch block after transcatheter aortic valve replacement is associated with adverse long-term clinical outcomes in intermediate-risk patients: an analysis from the PARTNER II trial. Eur Heart J. 2019;40:2218–27. 31505615 10.1093/eurheartj/ehz227

[21] Bennett M Parkash R Nery P. Canadian Cardiovascular Society/Canadian Heart Rhythm Society 2016 implantable cardioverter-defibrillator guidelines. Can J Cardiol. 2017;33:174–88. 28034580 10.1016/j.cjca.2016.09.009

[22] Meguro K Lellouche N Yamamoto M. Prognostic value of QRS duration after transcatheter aortic valve implantation for aortic stenosis using the CoreValve. Am J Cardiol. 2013;111:1778–83. 23528030 10.1016/j.amjcard.2013.02.032

[23] Roten L Meier B. Left bundle branch block after transcatheter aortic valve implantation: still a matter of concern? JACC Cardiovasc Interv. 2014;7:137–9. 24440019 10.1016/j.jcin.2013.11.006

[24] Dobson LE Musa TA Uddin A. The impact of trans-catheter aortic valve replacement induced left-bundle branch block on cardiac reverse remodeling. J Cardiovasc Magn Reson. 2017;19:22. 28222749 10.1186/s12968-017-0335-9 PMC5320804

[25] Eschalier R Massoullié G Nahli Y. New-Onset left bundle branch block after TAVI has a deleterious impact on left ventricular systolic function. Can J Cardiol. 2019;35:1386–93. 31492494 10.1016/j.cjca.2019.05.012

[26] Glikson M Nielsen JC Kronborg MB. 2021 ESC Guidelines on cardiac pacing and cardiac resynchronization therapy. Eur Heart J. 2021;42:3427–520. 34586378 10.1093/eurheartj/ehab699

[27] El-Menyar AA Abdou SM. Impact of left bundle branch block and activation pattern on the heart. Expert Rev Cardiovasc Ther. 2008;6:843–57. 18570622 10.1586/14779072.6.6.843

[28] Muntané-Carol G Urena M Nombela-Franco L. Arrhythmic burden in patients with new-onset persistent left bundle branch block after transcatheter aortic valve replacement: 2-year results of the MARE study. Europace. 2021;23:254–63. 33083813 10.1093/europace/euaa213

[29] Urena M Rodés-Cabau J. New-onset conduction disturbances: the last obstacle in the way of transcatheter aortic valve implantation. Eur Heart J. 2019;40:2228–30. 31071207 10.1093/eurheartj/ehz270

[30] Waksman R Khan JM. Left bundle branch block after TAVR: bubble or trouble? JACC Cardiovasc Interv. 2019;12:1185–7. 31129093 10.1016/j.jcin.2019.04.008

[31] Lenell J Lindahl B Karlsson P. Reliability of estimating left ventricular ejection fraction in clinical routine: a validation study of the SWEDEHEART registry. Clin Res Cardiol. 2023;112:68–74. 35581481 10.1007/s00392-022-02031-0 PMC9849182

